# The Time Course of Serum Iron and Serum Ferritin Concentrations Post‐COVID‐19 and Influenza Vaccinations

**DOI:** 10.1155/crdi/8662747

**Published:** 2026-06-28

**Authors:** Peter Lodemann, Oleg Tsuprykov, Rüdiger Lawaczeck

**Affiliations:** ^1^ Diamedikum Potsdam, Babelsberger Str. 28, 14473, Potsdam, Germany; ^2^ Faculty of Medicine, Institute of Mind, Brain and Behavior, HMU Health and Medical University, Olympischer Weg 1, 14471, Potsdam, Germany; ^3^ IFLB - Institute for Laboratory Medicine Berlin, Wohlrabedamm 8, 13629, Berlin, Germany; ^4^ Homeoffice Schulzendorf, Diakonieweg 7, 13503, Berlin, Germany

**Keywords:** COVID-19, mRNA vaccination timeline, SARS-CoV-2, serum ferritin, serum iron

## Abstract

**Introduction:**

Temporal responses of serum iron and ferritin in COVID‐19 infection and vaccination remain insufficiently characterized. This case report presents their timeline after SARS‐CoV‐2 and influenza vaccinations and may serve as a model for future studies. The approach can be extended to cohort studies or investigations of other acute‐phase reactants. A vaccination protocol with a defined timeline can provide a template for COVID‐19 and long COVID studies.

**Case Presentation:**

Blood samples were collected from vaccination through 6 weeks postvaccination, focusing on iron metabolism. Prevaccination values served as baseline controls. SARS‐CoV‐2 mRNA vaccination was administered together with routine seasonal influenza vaccination. Previous influenza vaccinations in this patient were not associated with systemic symptoms such as dizziness or fever; in contrast, the present case exhibited clear reactions, suggesting that the observed alterations in serum iron and ferritin are attributable to the SARS‐CoV‐2 vaccine. The vaccination induced an abrupt decrease in serum iron and a concomitant increase in ferritin. While ferritin returned to baseline within 6 weeks, iron levels steadily increased to approximately 1.8‐fold above baseline values but remaining within the reference interval.

**Discussion:**

The observed decrease in serum iron reflects an iron‐withholding response, a well‐established host defense mechanism during infections that limits pathogen proliferation. The increase in ferritin requires further interpretation. A hypothesis is presented, but further data are needed to support this mechanism. In future cohort studies, the protocol should include individual prevaccination values so that an additional control group is not necessary.

**Conclusion:**

Immune reactions to vaccines can trigger transient changes in serum iron and ferritin that resemble the acute‐phase response observed during infections. This case may serve as a template for studying the kinetics of immune responses, where *t* = 0 is defined by the vaccination time and each patient serves as own control when prior data are available.

## 1. Introduction

Long COVID‐19 patients exhibit a series of heterogeneous symptoms that can be attributed to prior SARS‐CoV‐2 infection. The range of symptoms includes chronic fatigue syndrome (CFS), weakness, and headache, as well as immune response reactions. Extensive studies have evaluated diagnostic symptoms and assessed risk factors [1–4]. Various parameters, such as severity of the infection, number of vaccinations, sex, and age, have been addressed. However, sex and age have not been analyzed in combination, although hormone and iron levels are interrelated, age‐dependent, and differ between men and women.

Studies on COVID‐19, including long‐ or post‐COVID‐19 cases, face a key limitation: the duration of infection or disease onset is often unknown, which interferes with the analysis of time‐dependent profiles and kinetics. The following case report on post–SARS‐CoV‐2 vaccination has two aims: (i) to establish and define a clear timeline and (ii) to increase the visibility of body iron levels throughout the infection period. Furthermore, SARS‐CoV‐2 vaccination can serve as a substitute for, or model of, real viral infections. The current mRNA‐based vaccination shares a key feature with viral infection in that a portion of the immune response is directed against the spike protein or its fragments [5]. The starting point of the immune response is clearly defined. The study relied on methods routinely used in general practice and on standard vaccination protocols funded by the general health insurance system.

## 2. Case Description

### 2.1. Patient

Male, 81‐year‐old, with age‐related health status, a non‐smoker, and a normal blood count, had persistent atrial fibrillation (ICD code I48.1) and was under treatment with bisoprolol 2.5 mg once daily and rivaroxaban 20 mg once daily. His arterial blood pressure was 120/85 mm Hg, and his BMI was 24.8 kg/m^2^. He received annual influenza vaccinations without noticeable adverse effects and followed semivegetarian diet.

The patient provided written informed consent for the publication of his data.

### 2.2. Vaccination and Hematological Parameters

Vaccination against the Omicron variant of the SARS‐CoV‐2 was administered using a 30 μg XBB.1.5 Comirnaty vaccine (Pfizer‐BioNTech), delivered intramuscularly into the left upper arm. This was the patient’s fifth dose of the BioNTech vaccine. Within the same session, the influenza vaccination was administered into the right upper arm. Both vaccinations were administered at 10:00 a.m. In the evening, the patient experienced an episode of severe chills (ague). The following morning, a routine annual check‐up, including blood count analysis, was performed. Blood was collected from the left cubital vein and was processed for routine blood analytes, including measurements of serum iron and ferritin. Blood samples were obtained in the fasting state. Blood sampling was repeated after 17 and 40 days. Routine blood test results from the preceding years were available and were used as reference data representing the patient’s pre–SARS‐CoV‐2 vaccination baseline.

**FIGURE 1:**
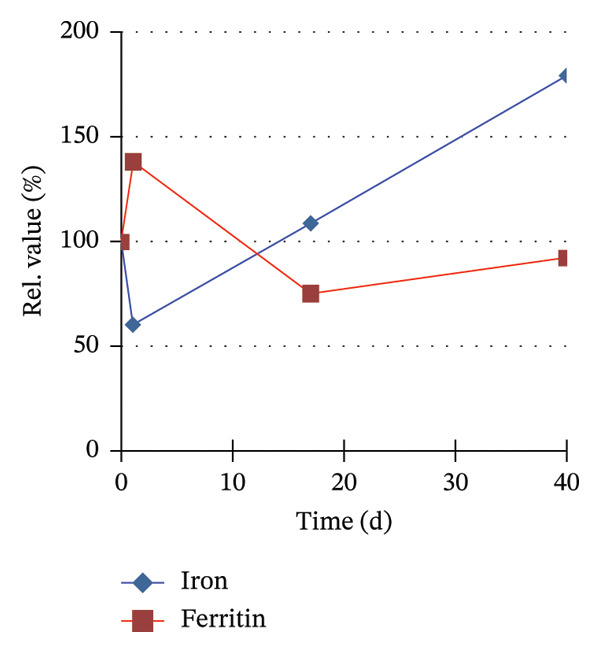
Serum iron and serum ferritin levels in percent (%) relative to the prevaccination serum iron and ferritin values (averaged over 4 years) of 88.3 ± 14.6 μg/dL and 65.2 ± 7.1 ng/mL, respectively, as function of time (d) postvaccination (*t* = 0).

The hematological parameters (hemoglobin, red blood cell [RBC] count, platelet count, and white blood cell [WBC] count), as well as serum iron and ferritin, are presented in Table 1 and shown for serum iron and ferritin levels in Figure 1 as relative changes from baseline or prevaccination value (shown values = actual value/baseline ∗ 100).

**TABLE 1:** Blood count values.

						**Mean/pre**	**Acute**	**Post-17**	**Post-40**
		**2019**	**2020**	**2021**	**2022**	**2023**	**2023**	**2023**	**2023**

Hemoglobin	g/dL	15.5	16.1	16.3	16	16	15.8		16.4
RBC	10^12^/L	5.41	5.47	5.5	5.42	5.45	5.45		5.56
Platelet count	10^9^/L	237	215	268	259	245	230		245
WBC	10^9^/L	7	7.3	7.2	8	7.4	7.4		7
Iron	μg/dL	83.9	111.4	71.1	86.8	88.3	53.6	96	158.4
Ferritin	ng/mL	56.2		73.6	65.7	65.2	90.0	49	60.2

*Note:* ‘Mean/pre‐2023’ refers to the mean values over 4 years; ‘acute’ refers to 24 h postvaccination; ‘post‐17’ and ‘post‐40’ refer to 17 and 40 days postvaccination, respectively. Blood tests were performed at the same laboratory, except for post‐17 days measurements in 2023. Acute phase markers (ACMs) were not included in the small blood test.

As can be seen in Figure 1, the serum iron concentrations decreased to a minimum shortly after vaccination, with a 24‐h value of 53.6 μg/dL, which is below the prevaccination values and below the reference interval (70 to 180 μg/dL). In the subsequent period of 6 weeks, the serum iron concentration continuously increases and reaches an unusually high level of 158.4 μg/dL on day 40, significantly above the prevaccination mean of 88.3 ± 14.6 μg/dL but still within the reference interval. Ferritin levels increased abruptly to a maximum of 90.0 ng/mL following vaccination and returned to normal (60.2 ng/mL) by day 40 postvaccination. For ferritin, a wide reference interval of 20 to 250 ng/mL is considered. Hemoglobin levels, RBC, platelet, and WBC counts remained within normal limits throughout the study period.

### 2.3. Laboratory Iron and Ferritin Tests

Serum iron and ferritin were measured colorimetrically on a Beckman Coulter AU5800 clinical chemistry analyzer (Beckman Coulter, Brea, CA, USA) using the iron OSR6286 (based on chemical reactions) and ferritin OSR61203 (based on antigen‐antibody reactions) reagents. Hemoglobin levels, RBC counts, WBC counts, and platelet counts were assessed on a Beckman Coulter DxH 900 (Miami, Florida, USA).

## 3. Discussion

### 3.1. Serum Iron and Ferritin

Serum iron and ferritin levels have been discussed in the context of SARS‐CoV‐2 infection and disease. In particular, sex‐specific effects can be interpreted in relation to the age‐ and sex‐dependent profiles of iron and ferritin [6]. Serum ferritin is upregulated during the immune response, independent of its role as a surrogate marker of the body’s iron status. Together with high ferritin levels (hyperferritinemia), reduced serum iron levels (hypoferremia) are considered prognostic markers of COVID‐19 outcomes [7–9]. For patients with long‐ or post‐COVID‐19, only a limited number of reports are available [2]; detailed longitudinal profiles over the course of therapeutic interventions are lacking, and the baseline time point (time zero) is rarely clearly defined. The Berlin Charité group [2] differentiated post‐COV/ME/CFS (post‐COVID/myalgic encephalomyelitis/CFS) and post‐COV/non‐ME/CFS from non‐COV/ME/CFS, with ferritin values of 90.9 ng/mL for the post‐COV/ME/CFS group and 69.9 ng/mL for the post‐COV/non‐ME/CFS group, which are comparable to the values observed in the present study (Table 1). The reported ferritin values are median values of the cohorts studied and can only be compared to reference values (20–300 ng/mL for males and 10–120 ng/mL for females), which are the internal reference intervals for ferritin accepted by the laboratory where both analytes were measured. Similarly to those of the Berlin Charité group, the Okayama/Japan group [10] reported decreasing ferritin levels from 193 ng/mL to 98.2 ng/mL and 86.7 ng/mL for the ME/CFS to non‐ME/CFS and to no‐fatigue groups, respectively [10]. Pasini et al. [11] reported large mean serum ferritin levels in long‐COVID patients at hospital admission and 60 days post‐discharge (following approximately 20 days of hospitalization) of 625 and 496 ng/mL, respectively, alongside parallel decreases in D‐dimer and CRP levels. A recent study (Doha/Paris) [12] confirmed an increase in ferritin levels correlating with the severity of long COVID‐19, accompanied by a slight decrease in high‐density lipoprotein cholesterol (HDL‐C). In 2024, the Cambridge group also presented results of its comprehensive study on iron dysregulation [13]. Following SARS‐CoV‐2 infection, serum iron levels are reduced, while ferritin and hepcidin are elevated. In long COVID patients normal values are reached over a longer period of months. The magnitude and duration of these changes appear to depend on the severity of symptoms. However, individual preinfection values and precise time‐zero reference are not available, and the temporal resolution during the early phase of infection is limited.

### 3.2. Ferritin and Transferrin

Ferritin is an intracellular iron storage protein complex composed of 24 H‐ and L‐chain proteins that assemble into a nanoscale nanocage capable of accommodating up to 4500 iron ions in the form of iron oxyhydroxides (see chemistry and biology of ferritin [14, 15]). The H‐ferritin chains not only constitute to the structural nanoscaffold but also possess oxidizing properties [16], catalyzing the oxidation of Fe^2+^ to Fe^3+^ (with electron release). Fe^3+^ represents the stored form of iron within the complex. The self‐assembly of ferritin subunits and its stepwise iron‐loading mechanism further highlight its potential for biotechnological application [17].

Serum ferritin, which undergoes glycosylation, is secreted from cells of the liver, spleen, kidneys and macrophages [16, 18, 19]. This effect can be attributed to acute‐phase reactants (APRs) or proteins (see recent review on the humoral innate immunity and acute‐phase proteins [20]). Serum ferritin levels have been combined with circulating C‐reactive protein, D‐dimer, and interleukin‐6 levels to determine the severity risk of COVID‐19 [21]. Circulating serum ferritin is generally accepted to belong to the APR family [20], but its physiological role remains to be fully elucidated. The vast majority of routine laboratory analyzers measure serum ferritin (total ferritin) without assessing the extent of iron stored within the ferritin complex.

The serum iron is tightly bound to transferrin, the iron transport protein in the blood. Free or labile serum iron is present in the circulation only in negligible amounts. In response to invading pathogens, the immune‐regulatory protein hepcidin is upregulated; it binds to ferroportin and thereby blocks any further iron supply (see the review by Mantovani and Garlanda [20]). A specific iron excretion pathway that regulates systemic iron levels has not been identified. In female rats, only approximately 10% of the radioactive Fe^59^‐labeled iron oxide nanoparticles were excreted into the feces within 42 days, with no detectable excretion via the urine, and in dogs no significant iron excretion was observed after 100 days [22]. Under normal conditions, iron homeostasis is regulated by controlling iron uptake [23], which is blocked via the hepcidin pathway as a consequence of the immune response. Hypothetically, ferritin might play a role in this context by binding iron released as debris from cellular immune reactions, thereby helping to maintain low serum iron levels. This double strategy may lead to reduced iron availability while maintaining relatively stable transferrin‐bound iron levels. Labile iron is a source of free radicals that can lead to tissue damage. Could serum ferritin play the role of a “*hoover”* for labile iron during the dynamic fluctuations (“the roller coaster ride”) of the immune response? If so, the role of serum ferritin as a scavenger for labile iron ions would complement that of haptoglobin and hemopexin, which are acute‐phase proteins that bind free hemoglobin and heme, respectively [20]. However, the answer to this hypothesis needs future investigations on serum ferritin and its iron loading.

In a published review on ferritin and inflammation [24], the role of ferritin as a biomarker in viral infections is discussed in detail, including early insights into COVID‐19, where it was already shown that serum ferritin is highly regulated during infection, consistent with the findings of the present case. However, a physiological explanation for the inflammation‐induced increase in serum ferritin was not provided and remains lacking.

The findings of the present case report warrant further in vitro and in vivo investigations. For human trials, most of the required methods are routinely available, and for individuals preparing for vaccination, the additional effort required to participate in such a cohort study should be minimal. Prevaccination values serve as individual controls, thereby eliminating the need for an additional control group.

## 4. Conclusions

In conclusion, this case report suggests that COVID‐19 mRNA vaccination is associated with an immune response characterized by decreased serum iron concentrations and increased ferritin levels. By 6 weeks postvaccination, these parameters returned to their prevaccination values. The timeline is well defined, in contrast to observations in COVID‐19 and long COVID‐19. Both serum iron and ferritin profiles can be interpreted as indicators of the body’s immune response related to iron metabolism, and they may help to improve our understanding of observations in COVID‐19 and long COVID‐19. Only routine laboratory tests, covered by the general health insurance system and performed on a daily basis, were used in the present study. The current case report can be extended to larger cohort studies. Prevaccination values serve as individual controls; an additional control group is unnecessary.

## Funding

This research did not receive any specific grant from funding agencies in the public, commercial, or not‐for‐profit sectors.

## Ethics Statement

The patient gave informed consent for the publication of this paper.

## Conflicts of Interest

The authors declare no conflicts of interest.

## Data Availability

The data that support the findings of this study are available upon request from the corresponding author. The data are not publicly available due to privacy or ethical restrictions.
